# Data on performance of air stripping tower- PAC integrated system for removing of odor, taste, dye and organic materials from drinking water-A case study in Saqqez, Iran

**DOI:** 10.1016/j.dib.2018.04.026

**Published:** 2018-04-12

**Authors:** Meghdad Pirsaheb, Jalil Mohammadi, Touba Khosravi, Hooshmnd Sharafi, Masoud Moradi

**Affiliations:** a Research Center for Environmental Determinants of Health, Kermanshah University of Medical Sciences, Kermanshah, Iran; b Water and Wastwater Company of Kurdistan, Sanandaj, Kurdistan Province, Iran; c Student Research Committee, Kermanshah University of Medical Sciences, Kermanshah, Iran

**Keywords:** Air stripping tower, PAC, Odor and Taste, Dye, Organic materials, Drinking water, Saqqez city

## Abstract

Unpleasant taste or smell are more importantly constituents of drinking-water, lead to complaints from consumers. Dye and organic matter as well change in disinfection practice may generate taste and an odorous compound in treated water. According to low efficiency of conventional methods to remove taste and odor compounds, present study was aimed to evaluate the performance of air stripping tower- Poly Aluminum Chloride (PAC) integrated system to remove odor and taste, dye and organic materials from drinking water. Different air to water ratio and PAC doses were used to remove considered parameters in certain condition. The results of this study indicated that the maximum removal efficiency of 86.2, 76.47, 58.46 and 41.27% of taste and odor, dye, COD and TOC were achieved by the air stripping tower- PAC integrated system, respectively. However, the physico-chemical characteristics of water and adsorbent effect on the of substances removal efficiency considerably. It can be stated that the air striping tower - PAC integrated system is able to reduce the odor and taste-causing substances and organic matter to a level which is recommended by the Institute of Standards and Industrial Research of Iran.

**Specifications table**

**Table:** 

Subject area	Environmental Health Engineering
More specific subject area	Environmental Chemistry
Type of data	Tables and figures
How data was acquired	The present study was to evaluate the performance of air stripping tower- PAC integrated system to remove odor and taste, dye and organic materials from drinking water. Initially a tower was built in type of packed and polyethylene glycol material with high working pressure (10 Atm), in 180 and 20 cm height and diameter dimensions, respectively. Different air to water ratio and PAC doses of16, 18, 20 and 22 mg/L were used in certain conditions to remove the considered parameters. In this study, a total of 57 samples were collected and the taste and odor, EC, pH, turbidity, color, TOC and COD were analyzed.
Data format	Raw, analyzed
Experimental factors	All samples were kept in polyethylene bottles in a dark place at room temperature.
Experimental features	The all above mentioned parameters were analyzed according to the standard method for water and wastewater treatment handbook.
Data source location	Saqqez, Kudistan province, Iran
Data accessibility	Data are included in this article

**Value of the data**

• Monitoring the quality of drinking water resources and trying to find efficient methods to remove of harmful pollutants, in term of human health, is necessary [1], [2], [3], [4], [5], [6], [7], [8], [9], [10].
• The drinking water consumer in Saqqez city suffer from odor and taste problems. Therefore, it is required to find the appropriate method to remove these problems.
• So far, there are no study on the effectiveness of this method to remove odor and taste from drinking water. Therefore, the data of this study could be an experience for future studies.
• The data of this study showed that the integrated method of air striping tower - PAC has high efficiency to reduce odor and taste-causing compounds and organic matter, consequently using of this method can afford frugality of energy (air striping) and PAC adsorbent consumption.

## 1 Data

Results indicated that the optimum ratio was [62.8/3.14 = 20] in the type 1 of air to water ratio, whereas the optimum of [188.4/62.8 = 3] was obtained for type 2. Also the removal of taste and odor, dye, TOC and COD for outlet water in the air stripping tower in (20 and 3) to 1 ratio of air to water were (82 and 61.38%), (68.7 and 76.47%), (23.32 and 13.73%) and (19 and 7.93%), respectively. However at integrated condition, the maximum removal efficiency (22 mg/L of PAC) of taste and odor, dye, TOC and COD for outlet water from the air stripping tower in (20 and 3) to 1 ratio of air to water were (86.2 and 86.2%), (76.47 and 76.47%), (58.46 and 45.68%) and (41.27 and 35.71%), respectively.

Accordingly, the removal rate of T & O-causing compounds increased with increased the amount of air ratio to water, which shows these are volatile compounds. Once the doses of PAC were used, the removal efficiency of taste and odor was increased.

The present study indicated that integrated method have a significant role in reducing the odor and taste compounds, dye, COD and TOC. Table 1 shows the quality of raw water (inlet) to air stripping tower. Table 2, Table 3 show the quality of outlet water of air stripping tower and quality of outlet water of air stripping tower follow the use of different PAC dosages, respectively.

**Table 1:** The quality of inlet water to air stripping tower.

**Parameters**	**Mean**	**SD**	**Min**	**Max**
Taste and odor (TON)	7.25	0.97	6	8.2
EC (µs/cm)	287	6.38	278	292
pH	8.06	0.08	7.95	8.12
Turbidity (NTU)	4.88	0.81	3.8	5.7
Dye (Hazen)	4.25	0.96	3	5
TOC (mg/L)	3.13	0.54	2.7	3.9
COD (mg/L)	12.6	0.54	11.9	12.7

**Table 2:** The quality of outlet water of air stripping tower in different air to water ratio.

**Parameters**	**Air to water ratio**	
	$\frac{62.8}{3.14}=20$	$\frac{188.4}{62.8}=3$
Taste and odor (TON)	1.3 ± 0.1	2.8 ± 0.15
EC (µs/cm)	278.3 ± 0.58	289.33 ± 2.08
pH	7.92 ± 0.01	7.93 ± 0.01
Turbidity (NTU)	1.97 ± 0.15	2.6 ± 0
Dye (Hazen)	1.33 ± 0.58	1.67 ± 0.58
TOC (mg/L)	2.4 ± 0	2.7 ± 0
COD (mg/L)	10.2 ± 0	11.6 ± 0

**Table 3:** The quality of outlet water of air stripping tower in different air to water ratio and PAC doses.

$\frac{air}{water}$	**Parameters**	**PAC dosages (mg/L)**			
		**16**	**18**	**20**	**22**
$\frac{62.8}{3.14}=20$	pH	7.8 ± 0.03	7.84 ± 0.01	7.83 ± 0.02	7.81 ± 0.05
	Turbidity (NTU)	1 ± 0.02	0.9 ± 0.03	0.7 ± 0.02	0.6 ± 0.04
	Taste and odor (TON)	1 ± 0	1 ± 0	1 ± 0	1 ± 0
	TOC (mg/L)	8.75 ± 0.05	7.9 ± 0.1	7.6 ± 0.07	7.4 ± 0.04
	COD (mg/L)	2.1 ± 0.1	2 ± 0	1.7 ± 0.03	1.3 ± 0.03
	Dye (Hazen)	1 ± 0	1 ± 0	1 ± 0	1 ± 0
$\frac{188.4}{62.8}=3$	pH	7.81 ± 0.03	7.82 ± 0.01	7.82 ± 0.02	7.83 ± 0
	Turbidity (NTU)	1.6 ± 0.1	1.3 ± 0.08	1 ± 0	0.8 ± 0
	Taste and odor (TON)	2.1 ± 0.1	1.3 ± 0.08	1 ± 0	1 ± 0
	TOC (mg/L)	10.3 ± 0.5	9.6 ± 0.3	8.9 ± 0.09	8.1 ± 0.06
	COD (mg/L)	2.5 ± 0.13	2.2 ± 0.1	1.8 ± 0.05	1.7 ± 0.04
	Dye (Hazen)	1 ± 0	1 ± 0	1 ± 0	1 ± 0

## 2 Experimental design, materials and methods

### 2.1 Design of air striping tower

**Fig. 1:**
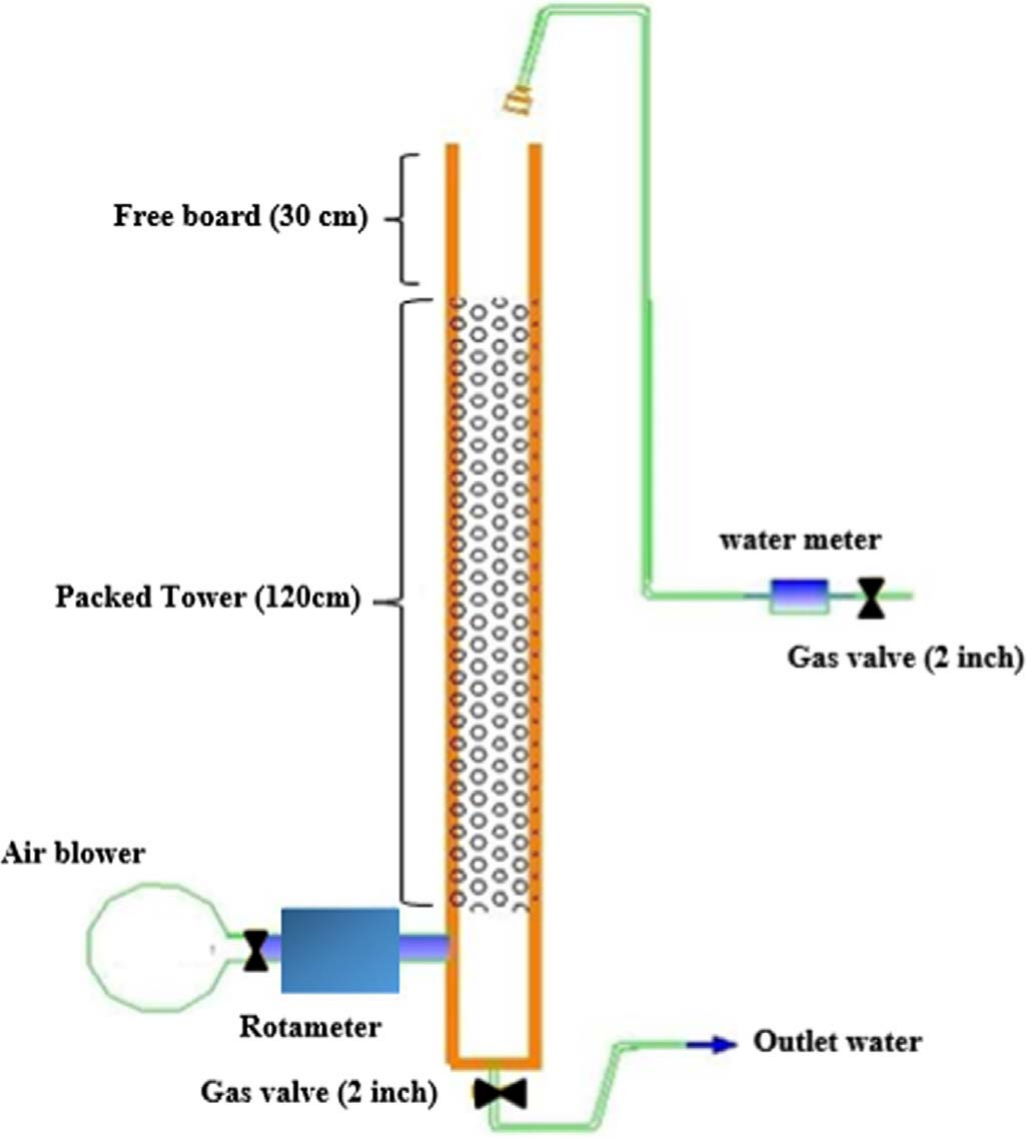
Schematic of air stripping tower.

A tower was built in type of packed and polyethylene glycol material with high working pressure (10 Atm), in 180 and 20 cm height and diameter dimensions, respectively. The roll made of polyethylene glycol was set in the column at height of 120 cm with 300 m^2^/m^3^ surface area (Fig. 1). The up and down of column was fixed by polyethylene mesh screen. Water was entered from up of the packed surface in the form of shower then collected by the pipe at bottom of column. Inlet Pipe with 30 cm length is also connected to the bottom of column. The pipe was designed as a siphon to prevent the outflow of air from outlet pipe, so that 5 cm of water head was over the pipe constantly. Then, the air stripping tower was installed at the inlet of water treatment plant of Saqqez. The required air for the tower was provided from existing air handling system of plant. In the present study, different ratio of air to water in two specific situations were used (the constant amount of water and variable air) and (the constant amount of air and variable water) (Table 4).

**Table 4:** Air-to-water ratios in two specific types (the constant amount of water and variable air) and (the constant amount of air and variable water).

**Conditions**		**Different air to water ratio**				
**Type 1**	The constant amount of water and variable air	$\frac{62.8}{3.14}=20$	$\frac{125.6}{3.14}=40$	$\frac{188.4}{3.14}=60$	$\frac{251.2}{3.14}=80$	$\frac{314}{3.14}=100$
**Type 2**	the constant amount of air and variable water	$\frac{188.4}{62.8}=3$	$\frac{188.4}{47.1}=4$	$\frac{188.4}{31.4}=6$	$\frac{188.4}{15.7}=12$	$\frac{188.4}{3.14}=60$

### 2.2 Method and material

Three samples were collected from the inlet water, then three samples were taken in each air to water ratio at different times (half of hour) in outlet of air stripping tower, so that 33 samples were taken at first step of study. At the end of each step, the ratio of air to water in inlet and outlet water of the air stripping tower was found, the most removal efficiency of T & O- causing compounds was selected as an optimum ratio. Follow that, the PAC doses of 16, 18, 20 and 22 mg/L were separately added to outlet water in the air stripping tower (at optimum air to water ratio) and subsequently jar test was determined. According to the four PAC mode doses, two optimum ratios of air to water, all 24 samples (three samples in each case) were taken and examined in the second step. The most effective and economical PAC was determined as follows: flock were settled after 20 min, the supernatant was filtered using filter paper, then due to the optimum air to water ratio, the certain PAC doses of16, 18, 20 and 22 mg/L were transferred to water obtained from previous stages and the optimum PAC dosage was determined. For all samples (57 samples), the taste and odor, EC, pH, turbidity, color, TOC and COD were examined. All samples were analyzed according to standard methods for water and waste water And other valid references [11], [12], [13], [14], [15], [16], [17], [18], [19].
