# Supplementary material for: Data on performance of air stripping tower- PAC integrated system for removing of odor, taste, dye and organic materials from drinking water-A case study in Saqqez, Iran
Source: Data Brief. 2018 Apr 12;18:1292–7. doi: 10.1016/j.dib.2018.04.026 (PMC5997578; doi:10.1016/j.dib.2018.04.026)
Supplement: Supplementary file 1 — Supplementary material [file mmc1.doc]

Dear editor(s)

Data in Brief

Greetings!

We would be grateful if you would consider the attached manuscript “**Data on performance of air stripping tower- PAC integrated system for removing of odor, taste, dye and organic materials from drinking water-A case study of Saqez city, Iran”** for publication in valuable Journal Data in Brief. We declare that this manuscript, or part of it, has neither been published nor is currently under consideration for publication by any other journal. We also declare that we have read the manuscript and approved submission to Journal of Data in Brief. The Project was supported by Water and Wastwater Company of Kurdistan, Kurdistan Province, Sanandaj, Iran. None of the authors of this paper have any conflicts of interest to declare.

Kind Regards,
